# The prognostic value of fat necrosis deposits on CT imaging in acute pancreatitis

**DOI:** 10.3906/sag-1910-31

**Published:** 2021-04-30

**Authors:** Şehnaz EVRİMLER, Münteha ÇAKMAKÇI, Adnan KARAİBRAHİMOĞLU, Mustafa KAYAN

**Affiliations:** 1 Department of Radiology, Faculty of Medicine, Süleyman Demirel University, Isparta Turkey; 2 Department of Biostatistics, Faculty of Medicine, Süleyman Demirel University, Isparta Turkey

**Keywords:** Computerized tomography, fat necrosis, pancreatitis

## Abstract

**Background/aim:**

Investigate the prognostic value of the fat necrosis deposit (FND) pattern in acute pancreatitis.

**Materials and methods:**

The contrast-enhanced computed tomography (CT) images of 35 necrotizing pancreatitis (NP) and 51 edematous pancreatitis (EP) cases were included in our retrospective study. Computed tomography severity index (CTSI) and Ranson scores were calculated. Images were evaluated for FND, complications (infection/ hemorrhage), walled-off necrosis (WON), and venous thrombosis (VT). We developed a new grading system called fat necrosis deposit-CTSI (FND-CTSI), which was the sum of FND and CTSI scores. The relationship between grading systems and mortality, length of hospital-intensive care unit stay, surgical and percutaneous interventions were evaluated.

**Results:**

FND-CTSI scores were significantly higher in NP than EP (P < 0.001). FND-CTSI demonstrated a significant correlation with CTSI (r:0.91, P < 0.001) and Ranson score (r:0.24, P = 0.025). CTSI was significantly higher in only mass form amongst the FND groups (P < 0.001). There was a significant difference in WON, complications, and mortality between FND groups (P < 0.05). CTSI and FND-CTSI scores were both significantly associated with WON, VT, surgical intervention, mortality (P < 0.001), and the presence of complications (P = 0.013 and P = 0.007, respectively). FND-CTSI was also significantly associated with percutaneous intervention (P = 0.019), while CTSI was not (P > 0.05). According to ROC analysis, AUC values of FND-CTSI were higher than CTSI for the detection of WON, complications, mortality, and percutaneous intervention (P < 0.05). FND-CTSI showed a highly significant correlation with the length of hospital and intensive care unit stays (P < 0.001).

**Conclusion:**

FND-CTSI can be used in acute pancreatitis grading and considered as a prognostic factor.

## 1. Introduction 

The prevalence of acute pancreatitis is estimated to be 10–50/10,000 and the mortality rate is approximately 6% [1–3]. Fatty tissue, fascia, and adjacent organs may also be affected in addition to the pancreas parenchyma in acute pancreatitis. Ranson [4], acute physiology and chronic health evaluation (APACHE II) [5], and CT severity index (CTSI) have been used as a clinical scoring system for the prognosis prediction of patients with acute pancreatitis for a long time [6,7].

Pancreatic necrosis generally appears homogeneous initially and later become heterogeneous by liquefication [8]. The severity of necrotizing pancreatitis (NP) depends on the necrosis ratios and is classified as <30%, 30%–50%, and >50%. Follow-up imaging is advised for patients with less than 30% necrosis which can resemble interstitial edematous pancreatitis [6]. Fat necrosis is a well-known complication of acute pancreatitis [9]. The most common localizations are peripancreatic fat tissue, omentum, and mesenteric fatty tissue [10]. Isolated peripancreatic necrosis is observed in less than 20% of cases, and these patients have a better prognosis than the ones with parenchymal necrosis [11]. Peripancreatic necrosis is considered when increased attenuation, linear stranding, and fluid collection is observed in peripancreatic fatty tissue. However, these findings can also be observed in acute interstitial edematous pancreatitis (EP). Heterogeneity in high attenuation suggests peripancreatic necrosis. A combination of both parenchymal necrosis and peripancreatic fat necrosis is the most common form of NP with a prevalence ranging between 75%–80% [8]. 

Fat necrosis can present as a peritoneal nodule, mesenteric implant, or mass in acute pancreatitis [12–14]. These findings may resemble peritoneal carcinomatosis or primary peritoneal cancer [14]. Therefore, it is suggested to evaluate patients with clinical findings and older examinations to avoid misinterpretation of these peritoneal and mesenteric lesions [15].

We observed fat necrosis deposit (FND) in patients who had severe pancreatitis in our daily practice. However, the FND pattern was different amongst the patients. Therefore, we aimed to investigate the prognostic value of the FND pattern in acute pancreatitis. 

## 2. Materials and methods

### 2.1. Study population and ethics

Acute pancreatitis was diagnosed according to the Revised Atlanta Classification System [16] in our study. A total of 86 patients were included, of which 35 (40.7%) were NP and 51 (59.3%) were EP. Informed consent was waived in our retrospective, and the institutional review board approved the study. 

### 2.2 Image acquisition and analysis

Images were performed on 128-sliced CT (Siemens Somatom Definition, Munich, Germany) scanner. Imaging parameters were as follows: tube voltage = 130 kV, effective mAs = 90, slice thickness 1mm, collimation = 2 × 4 mm, pitch = 1.6. Images were obtained at 70 s after intravenous administration of 100 mL Iopromide (Ultravist, Schering, Germany) at a speed of 3 mL/s. Images were evaluated with Picture Archiving and Communication System (PACS), OsiriX MA v 10.0.1 (UCLA, Pixmeo), GPL licensed free access resource code, and commercially licensed, FDA approved Mac OS X radiology work station.

**Figure 1:**
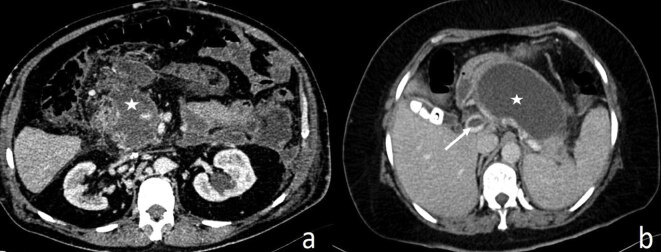
Walled-off necrosis (asterisk) surrounding portal vein (a) and walled-off necrosis (asterisk) reaching out into the portal vein (arrow) (b) are seen on the axial CT images of two different patients.

The abdominal CT images of the patients with acute pancreatitis performed 2–5 weeks after the disease onset were recruited because the liquefied components become more apparent after 1 week [8,11]. The images were evaluated independently by two radiologists Ş.E and M.Ç, who have 10 and 3 years of abdominal CT imaging experience, respectively. Each patient was evaluated according to the CTSI (Table 1) [17] and Ranson scoring system [4]. Images were also evaluated for complications (infection, hemorrhage), pseudocyst, walled-off necrosis (WON), and venous thrombosis (VT) (Figure 1a, 1b). Revised Atlanta Classification System classifies NP associated collections according to the disease onset time. Collections within 4 weeks without a wall are defined as the acute necrotic collection, whereas collections persisting after 4 weeks with an encapsulated wall are defined as WON [8, 16]. 

**Table 1:** Computed tomography severity index of acute pancreatitis and the sum of the scores of pancreatic inflammation and necrosis (mild acute pancreatitis = 0–3, moderate acute pancreatitis = 4–6, severe acute pancreatitis = 7–10) [6,17,26].

Pancreatic inflammation	Score
Normal pancreas	0
Focal or diffuse enlargement of the pancreas	1
Pancreatic +/- Peripancreatic inflammation	2
Single peripancreatic fluid	3
Two or more peripancreatic fluid+/-retroperitoneal air	4
Pancreatic necrosis	
None	0
< 30%	2
30%–50%	4
> 50%	6

**Figure 2:**
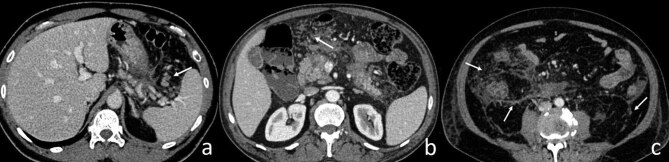
Peripancreatic FND (arrow) (a), anterior pararenal, and mesenteric FND (arrow) (b) are seen on the axial contrast-enhanced abdomen CT images of two different patients with necrotizing pancreatitis. FND localized at the paracolic area, lateroconal fascia, beyond Gerota’s fascia, and posterior renal fascia (arrows) are seen on the axial contrast-enhanced abdomen CT image (c).

**Figure 3:**
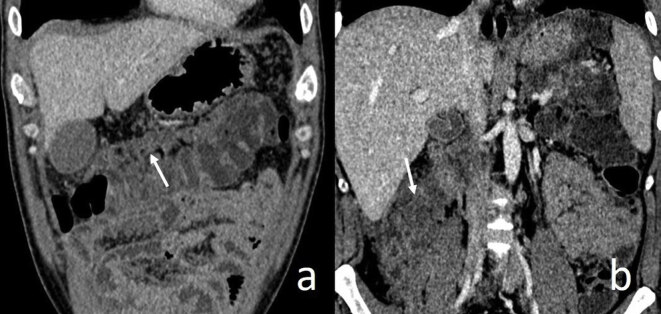
FND in mass form is seen at the transverse mesocolon resembling an omental cake (arrow) (a). Also, FND in mass form is seen close to the right psoas muscle on the coronal contrast-enhanced abdomen CT image (b).

The presence of Diabetes Mellitus (DM) was searched via electronic medical records. The scoring system according to the FND pattern was scored as follows; none:0, only peripancreatic:2 (Figure 2a), anterior pararenal/mesenteric:4 (Figure 2b), beyond Gerota’s fascia:6 (Figure 2c), and mass form:8 (Figure 3a, 3b). Pancreatic necrosis in CTSI and pancreatic-peripancreatic necrosis in Modified CTSI are defined by ordered even numbers [7]. Therefore, we used ordered even numbers in our FND scoring system which also enables statistical calculations. In addition, we developed a new grading system called Fat Necrosis- CT Severity Index (FND-CTSI) as the sum of FND and CTSI scores. Mortality, duration of hospital stay (≤25 days, >25 days), duration in the intensive care unit (≤1 day, >1 day), surgical (none, cholecystectomy, pancreaticojejunostomy, Roux en-y cystojejunostomy, pancreatic cyst surgical external drainage), and percutaneous interventions were evaluated as prognostic factors. 

### 2.3. Statistical analysis

Statistical analyses of the study were performed by SPSS (IBM Inc., Chicago, IL, USA) with version 20.0. Power analysis was performed by GPower software (Ver 3.1.9.2, Kiel, Germany). The number of patients required for the study was determined by power analysis for Chi-square analysis of NP and EP groups with 0.30 effect size, 5% error, and 80% power. Groups were determined by the single blinding method. Descriptive statistics were presented as frequencies (percentage) for categorical variables; as median and interquartile range (IQR) for numerical variables. Continuous variables were analyzed for normality by the Kolmogorov-Smirnov test. The associations between variables amongst categorical data were determined by the corrected Chi-square analysis. Since the distributions of the measurements were not normal, the Mann-Whitney U test for two independent samples and the Kruskal-Wallis test for multiple samples were used for group comparison. Next, pairwise comparisons with Bonferroni correction for multiple testing were performed. Receiver Operating Characteristic (ROC) curve analysis was done to examine the performance of CTSI and FND-CTSI using Area Under the Curve (AUC). A p-value of less than 0.05 (P < 0.05) was considered statistically significant by taking 5% for type-I error.

## 3. Results

The mean age was 51.78±13.85 and 56.74±16.63 in the NP and EP group, respectively. The percentage of male and female patients were 52.3 and 47.7, respectively. There was no significant relationship in gender distribution and mean age between NP and EP groups (P = 0.473 and P = 0.081, respectively). 

Pseudocyst was only observed in EP (9/51) (17.7%), WON was only observed in the NP group (29/35) (82.8%). WON was observed in the intraparenchymal and peripancreatic distribution in 86.5%, intraparenchymal in 8.1%, and peripancreatic in 5.4%. VT was observed only in the NP group (9/35) (25.7%) (P < 0.001). There was no significant difference in the presence of complications (P = 0.07) and DM (P = 0.69) between the groups. 

Inter-reader reliability for FND-CTSI was calculated as excellent (ICC:0.94, 95% Confidence Interval: 0.87-0.97, P < 0.001). FND distribution was as follows; none (n=60), only peripancreatic (n=6), anterior pararenal/mesenteric (n=12), beyond Gerota’s fascia (n=3), and mass form (n=5). The ratio of the presence of FND in the NP group was 26/35 (74.29%). CTSI was significantly higher in only mass form FND group (P < 0.001), but there was no significant difference between the other groups (P > 0.05). FND-CTSI scores exhibited a compatible increase with FND distribution (P < 0.001). There was a significant difference in WON (P = 0.05), complications (P = 0.004), and mortality (P = 0.006) between the FND groups. The rate of complications and mortality was observed more in the mass form group, while WON was more in the beyond Gerota’s fascia group. 

CTSI and FND-CTSI were significantly higher in NP (6.60±2.25 and 9.91±4.05, respectively) group than the EP group (2.75±0.74 and 2.74±0.75, respectively) (P < 0.001) *.* There was no significant difference in the Ranson score (NP:1.89±1.04; EP:1.62±1.31) between the groups (P = 0.18) *.* FND-CTSI demonstrated a significant and very strong correlation with CTSI (r:0.91, P < 0.001). FND-CTSI demonstrated a significant and weak correlation with Ranson (r:0.24, P = 0.025). 

FND-CTSI was significantly associated with necrosis ratio (P < 0.001). CTSI and FND-CTSI scores were both significantly associated with WON, VT, surgical intervention, and mortality (P < 0.001). CTSI and FND-CTSI scores were also significantly associated with the presence of complications (P = 0.013 and P = 0.007, respectively). FND-CTSI was significantly associated with percutaneous intervention (P = 0.019), while CTSI was not (P > 0.05). None of the scores were significantly associated with DM (P > 0.05) (Table 2). 

**Table 2:** The relationship between computed tomography severity index, Fat necrosis deposit-computed tomography severity index and walled-off necrosis, venous thrombosis, complication, diabetes mellitus, mortality, percutaneous interventions, surgical interventions.

		CTSI Median- IQR	p	FND-CTSI Median-IQR	P
WON	Negative (n = 57)	3 (2)	<0.001*	3 (2)	<0.001*
	Positive (n = 29)	6 (4)		10 (6)	
VT	Negative (n = 77)	3 (3)	<0.001*	3 (6)	<0.001*
	Positive (n = 9)	10 (3)		11 (7)	
Complication	None (n = 81)	3 (3)	0.013*	3 (6)	0.007*
	Infection/Hemorrhage (n = 5)	8.5 (6)		14.5 (12)	
DM	Negative (n = 75)	4 (3)	0.79	4 (6)	0.77
	Positive (n = 11)	3 (7)		3 (7)	
Mortality	Alive (n = 71)	3 (2)	<0.001*	3 (3)	<0.001*
	Ex (n = 5)	9 (3)		17 (4)	
Percutaneous intervention	None (n = 59)	3 (3)	0.07	3 (6)	0.019*
	Abscess/Ascites/Effusion drainage (n = 3)	6 (.)		12 (.)	
Surgical intervention	None (n = 50)	3 (2)	<0.001*	3 (2)	<0.001*
	Positive (n = 13)	3 (4)		11 (8)	

According to ROC analysis, AUC values of CTSI and FND-CTSI for WON, VT, complication, mortality, and surgical intervention were statistically significant (P < 0.05), but not for DM. FND-CTSI showed higher AUC for the detection of WON, complication, mortality, and percutaneous intervention whereas CTSI showed higher AUC for the detection of VT and surgical intervention (Table 3).

**Table 3:** ROC analysis of CTSI and FND-CTSI for walled-off necrosis, venous thrombosis, complication, and surgical intervention.

ROC analysis		AUC	95% Confidence Interval	p	Cut-off	Sensitivity	Specificity
WON	CTS IFND-CTSI	0.96 0.99	0.93-1.00 0.97-1.00	<0.001* <0.001*	4.5 8.5	89.7 72.41	65 100
VT	CTSIFND-CTSI	0.940.93	0.86-1.000.87-1.00	<0.001*<0.001*	6.59.5	88.8988.89	90.0990.09
COMPLICATION	CTSI FND-CTSI	0.83 0.86	0.63-1.00 0.64-1.00	0.015* 0.008*	7.5 12.5	80.00 80.00	88.9 95.1
MORTALITY	CTS IFND-CTSI	0.95 0.99	0.90-1.00 0.97-1.00	<0.001* <0.001*	6.5 15	80.00 80.00	91.5 98.6
SURGICAL INTERVENTION	CTS IFND-CTSI	0.92 0.90	0.83-1.00 0.80-0.99	<0.001* <0.001*	4.5 6.5	84.6 92.3	82 82
PERCUTANEOUS INTERVENTION	CTS IFND-CTSI	0.81 0.88	0.63-0.99 0.75-1.00	0.07 0.026 *	8.5	66.7	78

CTSI and FND-CTSI scores were both significantly higher in the groups with longer duration of hospital stay and duration in the intensive care unit (P < 0.001) (Table 4).** According to cox regression analysis, both scoring systems showed a highly significant and positive correlation with the length of hospital (CTSI; r:0.51, P < 0.001 and FND-CTSI; r:0.53, P < 0.001) and intensive care unit (CTSI; r:0.58, P < 0.001 and FND-CTSI; r:0.57, P < 0.001) stays.**

**Table 4:** The relationships between CTSI and FND-CTSI scores and length of hospital stay and intensive care unit stay.

	Hospital Stay		
	≤25 day (n = 48)Median (IQR)	>25 day (n = 14)Median (IQR)	P
CTSI	3 (2)	6.5 (4)	<0.001*
FND-CTSI	3 (2)	9 (5)	<0.001*
	Intensive care unit stay		
	≤1 day (n = 50)	>1 day (n = 12)	P
CTSI	3 (2)	8 (3)	<0.001*
FND-CTSI	3 (3)	11 (9)	<0.001*

## 4. Discussion

FND-CTSI is a newly developed CT grading, which is composed of CTSI and FND scores. To the best of our knowledge, this is the first study to investigate the value of FND in CT grading of acute pancreatitis. There was a significant difference in WON, complications, and mortality between FND groups. FND-CTSI was significantly associated with necrosis ratio, WON, VT, complication, mortality, and a need for surgical or percutaneous intervention. It also showed a highly significant and positive correlation with the length of hospital and intensive care unit stays.

Intraabdominal fat is located in both of the retroperitoneal and intraperitoneal compartments [18,19]. Fat necrosis is a known complication of acute pancreatitis [9]. The most common localizations are peripancreatic fat tissue, omentum, transverse mesocolon, and mesentery [10,20]. Although retroperitoneum is a common localization for pancreatitis and fat necrosis, it is generally limited within anterior and posterior pararenal spaces. It is suggested that fat necrosis is caused by the discharge of lipase into the lymphatic and vascular system during acute pancreatitis [10]. Fat necrosis occurs as a result of fat saponification by released lipolytic enzymes from the affected parenchyma [17,21,22]. The affected fat tissue activates the macrophage and other inflammatory mediators and aggravates the inflammatory response [17,22]. Phospholipase and protease attack the plasma membrane of fat cells, which release triglycerides. Free fatty acids are formed by hydrolysis. Then, calcium soaps are formed by the combination of fatty acids and calcification. This theory is suggested as the reason for hypocalcemia in severe pancreatitis. Fat necrosis deposits are distributed in the retroperitoneum and abdominal cavity after the resolution of acute exudate and ascites [23]. These nodules may cause mass effect and late enhancement because of the slow diffusion of contrast material from the capillaries in granulation tissue [13,24]. The extrapancreatic spread of exudate is a common complication of pancreatitis. It is generally observed in retroperitoneum and anterior pararenal spaces but doesn’t spread beyond renal fascia. Thus, kidneys and perirenal fat tissue are not affected [19]. On the other hand, there are cases of subcapsular and intrarenal pancreatic fluid collections in the literature [25]. Gerota’s fascia is thought to act as a barrier between pararenal and perirenal spaces [24]. In our study, most of the NP cases had FND and the majority of them were distributed in the anterior pararenal space. However, we also observed patients with FND distributed beyond Gerota’s fascia. 

In our study, there were also some patients whose FND was in mass form. Fat necrosis can also present as a gross palpable anterior abdominal mass [12]. Pedrosa et al. presented a case of a renal pseudotumor caused by retroperitoneal fat necrosis secondary to acute pancreatitis. Fat necrosis should be kept in mind for the differential diagnosis of renal tumors in patients with acute pancreatitis. Progressive and prolonged contrast enhancement can help to identify the pseudotumor in such cases [13]. Smith et al. described fluid collections, solid enhancing peritoneal nodules, mesenteric implants, and bulky soft tissue lesion surrounding vascular structures at the mesenteric root. They interpreted these findings as a result of exuberant granulomatous reactions secondary to acute pancreatitis. The findings resembled peritoneal carcinomatosis or primary peritoneal carcinoma [14]. Therefore, it is necessary to differentiate nodular fat necrosis from peritoneal malignancies by evaluating clinical-laboratory findings and previous imaging findings showing pancreatitis [15]. The CT findings of some of the patients in our study were also seemed to be a mimicker of peritoneal carcinomatosis.

Inflammation, pancreatic necrosis, and local complications are the evaluated factors to differentiate between mild acute pancreatitis (interstitial/edematous) and severe pancreatitis (necrotizing). This enables to implement the precise treatment management [6,26]. In our study, both CTSI and newly developed FND-CTSI scores were higher in NP than EP. FND-CTSI demonstrated a significant correlation with both CTSI and Ranson scores. CTSI was significantly higher in only mass form amongst the FND groups.

Extrapancreatic complications such as pleural effusion, ascites, vascular complication (venous thrombosis, arterial hemorrhage, pseudoaneurysm), parenchymal complication (infarction, hemorrhage, subcapsular fluid collection), and gastrointestinal involvement (inflammation, perforation, intraluminal fluid collection) were evaluated in addition to the pancreatic findings to determine the severity of acute pancreatitis in the previous studies [7,27,28]. However, none of these studies evaluated the FND pattern. An increase in the FND score resulted in a significant increase in complications and mortality. We observed that both FND-CTSI and CTSI were significantly higher in patients who needed surgical intervention, similar to the studies in the literature. Both scores were also significantly associated with WON, VT, complication, and mortality. FND-CTSI was also significantly associated with percutaneous intervention, while CTSI was not. 

Balthazar et al. observed an excellent correlation between CTSI and necrosis, duration of hospital stay, complication development, and death [6,26]. CTSI and pancreatic necrosis were significantly associated with the need for intervention [29]. Leung et al. declared that CTSI was more sensitive than the Ranson score in the prediction of complication, mortality, and duration of hospital stay [30]. FND-CTSI provided better diagnostic performance for the detection of WON, complication, mortality, and percutaneous intervention, whereas CTSI showed better diagnostic performance for the detection of VT and surgical intervention in the current study. CTSI and FND-CTSI had a significant association and correlation with the length of hospital and intensive care unit stay in our study similarly with the literature.

The limitations of our study are as follows: limitations adherent to the retrospective design; we could only evaluate the prognosis of the patients that we could manage the information from the electronic medical records, small patient population.

## 5. Conclusion

Acute pancreatitis can cause fat necrosis. Fat necrosis deposits are primarily seen in necrotizing pancreatitis. FND-CTSI is associated with the severity of disease and prognosis. Therefore, we suggest that FND-CTSI can be used in acute pancreatitis grading, and the grade of FND pattern may be a prognostic factor that can help with clinical decision making regarding the treatment.
